# Acute methotrexate-induced cutaneous ulceration in generalized pustular psoriasis: A case report and review of the literature

**DOI:** 10.1016/j.jdcr.2025.10.068

**Published:** 2025-11-13

**Authors:** Mazin Aljabri, Luai Assaedi, Shumukh Alqahtani, Ashwaq Al-Osaimi, Aishah Radin, Walaa Ahmed

**Affiliations:** a Department of Dermatology, Heraa General Hospital, Makkah, Kingdom of Saudi Arabia; b Department of Dermatology, Makkah Health Cluster, Makkah, Kingdom of Saudi Arabia

**Keywords:** generalized pustular psoriasis, methotrexate, methotrexate-induced cutaneous ulceration

## Introduction

Generalized pustular psoriasis (GPP) is a rare, severe form of psoriasis characterized by widespread sterile pustules on an erythematous base, often with systemic symptoms.^1^ Unlike chronic plaque psoriasis, GPP can be life-threatening and lacks standardized diagnostic criteria, making clinical distinction from conditions like acute generalized exanthematous pustulosis essential.^2^

Methotrexate (MTX) is a commonly used systemic therapy for psoriasis, including GPP, due to its anti-inflammatory and immunosuppressive properties.^3^ However, MTX toxicity can lead to mucocutaneous ulceration, hepatotoxicity, and bone marrow suppression.^3^ Cutaneous ulceration in particular, although well documented in plaque psoriasis, has not been reported in the context of GPP.^4^

We report a rare case of MTX-induced cutaneous ulceration in a patient with GPP. To our knowledge, this is the first such case documented in the literature. This report highlights the importance of monitoring for toxicity even at therapeutic doses and explores the role of targeted therapies such as spesolimab in managing refractory GPP. This case also provides visual documentation of GPP and MTX toxicity in skin of color, which is under-represented in the literature.

## Case presentation

A 34-year-old woman with a history of GPP, first diagnosed during her third trimester in 2018, presented in June 2024 with widespread erythematous plaques and pustules. Diagnosis was confirmed via skin biopsy showing orthokeratosis, a diminished granular layer, and dermal perivascular inflammatory infiltrates with neutrophils and eosinophils. At the time of her initial presentation in 2018, she responded well to systemic corticosteroids and cyclosporine and achieved full remission postpartum with oral MTX 10 mg weekly for 5 months, followed by topical maintenance.

**Fig 1:**
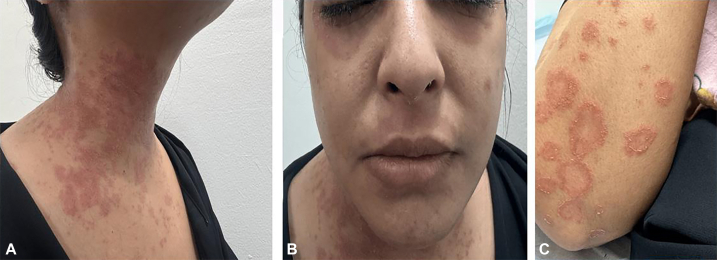
Clinical presentation of the patient before initiation of methotrexate (MTX). **A** and **B,** Active erythematous plaques with peripheral pustules on the neck and upper chest. **C,** Erythematous plaques with pustules on the forearm.

In 2020, she had a mild recurrence during her second pregnancy, managed with topical therapy alone. She remained stable until June 2024, when she presented with a severe flare of GPP accompanied by fever. There was no history of nonsteroidal anti-inflammatory drug use prior to the flare. Intravenous (IV) hydrocortisone was given by emergency physician but led to worsening of her condition. She was subsequently initiated on MTX 15 mg weekly with daily folic acid (5 mg). Baseline laboratory testing was performed prior to reinitiating MTX, and weekly complete blood count and metabolic panels were obtained during treatment. At that time, she exhibited active erythematous plaques with peripheral pustules on the neck, chest, and forearms (Fig 1).

**Fig 2:**
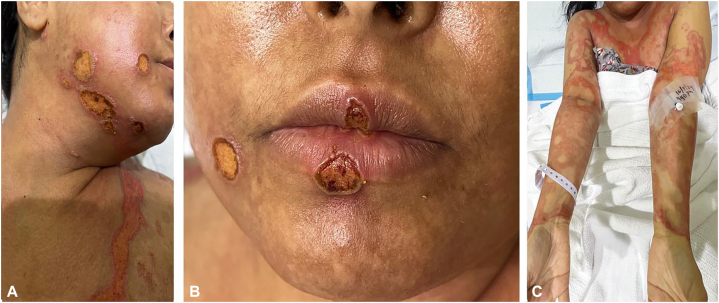
Clinical presentation of the patient after 3 doses of oral weekly methotrexate, showcasing signs of MTX-induced cutaneous ulceration. **A,** Ulceration of previous psoriatic lesions on the right jaw and upper chest. **B,** New ulcerative lesions on the lips. **C,** Extensive erosions and erythematous plaques on the upper extremities. *MTX*, Methotrexate.

After 3 doses of MTX, the patient developed new annular ulcerations on the lips and erosions over pre-existing plaques, sparing the scalp, nails, palms, and soles (Fig 2). There were no intraoral ulcerations; lesions were limited to the lips. Laboratory investigations revealed hepatotoxicity with alanine aminotransferase at 902 U/L and aspartate aminotransferase at 210 U/L; white blood cell count was 2.8 × 10^9^/L, absolute neutrophil count was 1.1 × 10^9^/L, hemoglobin dropped from 14.9 to 11.7 g/dL, then to 9.2 g/dL, and platelet count decreased from 427 to 250 × 10^9^/L. Albumin was 27 g/L, blood urea nitrogen was 8.5 mmol/L, and estimated glomerular filtration rate was 78 mL/min/1.73 m^2^.

MTX was discontinued, and she was admitted with suspected toxicity. At that time, serum MTX levels were not available. She received IV leucovorin (20 mg every 6 hours for 4 days), IV albumin, local wound care, and supportive measures. A wound culture from the jaw grew methicillin-resistant *Staphylococcus aureus* and a nasal swab confirmed colonization. IV vancomycin was initiated. After four days of leucovorin therapy, the MTX level was measured and found to be undetectable at the time of testing. Despite improvement in liver function, her hemoglobin declined further, and a workup revealed vitamin B12 deficiency, which was treated with subcutaneous cyanocobalamin and oral folic acid.

**Fig 3:**
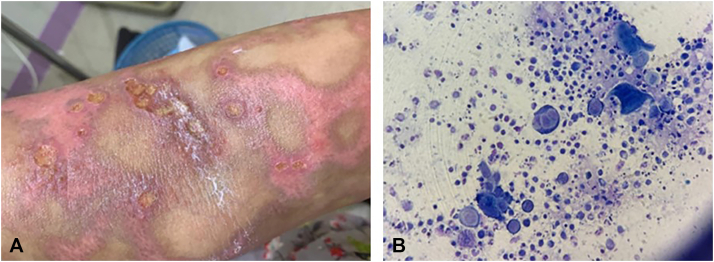
Clinical and microbiological findings during admission, highlighting superinfection with herpes simplex virus (HSV). **A,** New punched-out erosions and vesicular lesions with an erythematous base on the arm. **B,** Tzanck smear showing multinucleated giant cells, consistent with HSV superinfection.

At the time of initial ulceration, herpes simplex virus (HSV) testing was not performed. However, on the fourth day of hospitalization, new painful vesicular lesions and punched-out erosions developed in the axillae and antecubital fossae, accompanied by fever spikes (Fig 3, *A*). A Tzanck smear confirmed HSV superinfection, showing multinucleated giant cells (Fig 3, *B*). She was treated with IV and topical acyclovir, leading to resolution of lesions. The patient was discharged on day 9 with ongoing topical therapy.

**Fig 4:**
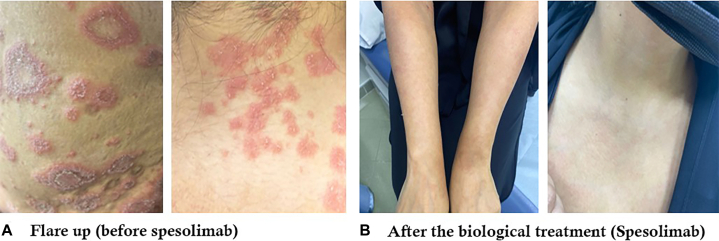
Clinical presentation of the patient during a generalized pustular psoriasis (GPP) flare and after treatment with spesolimab. **A,** Active erythematous plaques with pustules before spesolimab treatment. **B,** Significant resolution of lesions following spesolimab therapy.

At 12-week follow-up, she remained lesion-free. However, at 16 weeks, she experienced a nonfebrile GPP flare characterized by widespread erythematous plaques and pustules. A single IV dose of spesolimab, an interleukin-36 receptor antagonist, led to near-complete resolution of pustules within 24 hours (Fig 4).

## Discussion

MTX is a widely used systemic immunosuppressant for psoriasis and is generally well tolerated at weekly doses of 5 to 15 mg. However, MTX toxicity can still occur even within therapeutic ranges, affecting multiple organ systems and manifesting as hepatotoxicity, pancytopenia, mucositis, and cutaneous ulcerations. While gastrointestinal symptoms are commonly observed, mucocutaneous ulcerations remain a rare but severe adverse event.^5^

Cutaneous ulceration associated with MTX is most frequently reported in patients with psoriasis, although cases have also been noted in atopic dermatitis, bullous pemphigoid, and mycosis fungoides.^6^ In a case described by Boyd and Menter, a 58-year-old man developed multiple mucocutaneous ulcers after 32 weeks of 15 mg/week MTX, which resolved after cessation of therapy.^6^ Berna et al reviewed 114 similar cases and found that psoriasis was the most common underlying condition (69.3%) and that renal dysfunction (37.8%), inadequate folic acid supplementation (89.1%), and dosing errors (30%) were key contributors to toxicity. Alarmingly, 12% of patients died, mostly due to renal impairment or pancytopenia.^7^

In our case, the patient developed painful erosive and ulcerative lesions while on a stable dose of 15 mg/week MTX despite taking 5 mg of folic acid daily. She had no known comorbidities, history of dosing error, or concomitant medications known to interact with MTX, such as nonsteroidal anti-inflammatory drugs, trimethoprim/sulfamethoxazole, amoxicillin, or dapsone. Notably, ulcerations did not localize within psoriatic plaques, as is more typical in MTX-induced ulcerations in psoriasis. Instead, they appeared on nonlesional skin and mucosa, which may suggest a systemic toxicity mechanism rather than a local Koebner-type reaction.

Despite the absence of typical risk factors, several alternative contributors were identified. Her low serum albumin may have led to increased free MTX levels by reducing protein binding, and her low vitamin B12 level could have impaired DNA synthesis, compounding hematologic toxicity. Mild renal dysfunction may have further reduced MTX clearance. Additionally, concurrent infections with methicillin-resistant *Staphylococcus aureus* and HSV could have amplified systemic inflammation, potentially exacerbating MTX-induced ulceration and prolonging recovery.

The patient’s clinical course was further complicated by pancytopenia and elevated liver enzymes. MTX was promptly discontinued, leucovorin rescue therapy was initiated, and comprehensive laboratories including complete blood count and metabolic panel were monitored serially. Supportive care and antimicrobial therapy were essential given the superinfections. This management strategy aligns with standard MTX toxicity protocols and underscores the need for rapid recognition and intervention.

This case highlights the unpredictable nature of MTX toxicity and the importance of individualized risk assessment. Clinicians must monitor laboratory parameters closely, especially in patients with pustular psoriasis subtypes like GPP. Although MTX remains a therapeutic option for GPP, newer agents such as interleukin-36 receptor antagonists offer promising alternatives. In this case, spesolimab induced rapid pustular clearance during a subsequent flare, suggesting its efficacy in refractory GPP. Ultimately, clinicians should remain alert to early signs of MTX toxicity and consider biologic therapy when appropriate.

## Conclusion

This case emphasizes that MTX toxicity can develop abruptly in GPP patients, even at standard doses and with prior tolerance. Clinicians should maintain a high index of suspicion for early signs of mucocutaneous toxicity and regularly monitor hematologic and hepatic parameters. Targeted therapies like spesolimab may offer effective and safer alternatives in refractory or high-risk patients, potentially redefining treatment strategies in severe GPP.

## Conflicts of interest

None disclosed.
